# Effect of Exogenous Gibberellin, Paclobutrazol, Abscisic Acid, and Ethrel Application on Bulblet Development in *Lycoris radiata*

**DOI:** 10.3389/fpls.2020.615547

**Published:** 2021-01-20

**Authors:** Junxu Xu, Qingzhu Li, Ye Li, Liuyan Yang, Yongchun Zhang, Youming Cai

**Affiliations:** ^1^Forestry and Pomology Research Institute, Shanghai Academy of Agricultural Sciences, Shanghai, China; ^2^Agricultural Technology Extension Service Station of Langxia Town, Shanghai, China

**Keywords:** Lycoris radiata, bulblet development, hormone application, carbohydrate metabolism, regulation

## Abstract

*Lycoris* species have great ornamental and medicinal values; however, their low regeneration efficiency significantly restricts their commercial production. Exogenous hormone application is an effective way to promote bulblet development, but their effect on *Lycoris radiata* has not been verified to date. In the present study, we examined the effect of different exogenous hormones on bulblet development in *L. radiata*, and found that gibberellic acid (GA) significantly inhibited, whereas paclobutrazol (PBZ), abscisic acid (ABA), and ethrel promoted bulblet development, especially PBZ, a GA biosynthesis inhibitor. Furthermore, GA reduced endogenous cytokinin (CK) content, as well as the activities of carbohydrate metabolism enzymes, including sucrose synthase (SUS) and glucose-1-phosphate adenylyltransferase (AGPase), by downregulating the expression levels of *LrSUS1*, *LrSUS2*, and genes encoding AGPase large and small subunits. This resulted in the decrease in carbohydrate accumulation in the bulblets, thus hindering their development. PBZ had the opposite effect to GA on carbohydrate metabolism; it decreased endogenous GA_15_ and GA_24_, thereby promoting bulblet development. ABA promoted endogenous auxin content and the activities of starch synthesis enzymes, especially soluble starch synthase (SSS) and granule-bound SS (GBSS), through the up-regulation of the expression levels of *LrSS1*, *LrSS2*, and *LrGBSS1* genes, which could also result in the accumulation of carbohydrates in the bulblets and promote their development. In addition, ethrel application partly promoted bulblet development by promoting endogenous CK content. Although the accumulation of carbohydrates and the activity of starch enzymes were increased by ethrel treatment, we hypothesized that the effect of ethrel on regulating carbohydrate metabolism may be indirect. Our results could provide a basis for improving the propagation efficiency of *L. radiata* for production, as well as propose some directions for future research.

## Introduction

Species from the genus *Lycoris*, which are mainly distributed in southwestern China and Japan (Shi et al., 2006), have great ornamental and medicinal values. The colors of *Lycoris* flowers are diverse; for example, the flowers of *Lycoris longituba* commonly appear in several distinct colors: white, purple, red, orange, and yellow (He et al., 2011). *Lycoris* species also have great potential for commercial use, as Amaryllidaceae alkaloids isolated from their bulbs have been proven to have analgesic, anti-inflammatory, antiviral, anti-malarial, anti-tumor, and antineoplastic activities (Jin, 2009). The demand for *Lycoris* bulbs has increased in recent years; however, their natural regeneration efficiency is very low. As a result, the overexploitation of their natural habitats has led to a significant decrease in wild *Lycoris* resources (Chang et al., 2013). Thus, improving the reproductive efficiency of *Lycoris* species would be beneficial for both the protection of wild resources and the development of *Lycoris* commercial use.

The reproductive efficiency of *Lycoris* species is mainly determined by their bulblet propagation efficiency (Xu et al., 2020). Previous studies have shown that in *Lycoris sprengeri* and *Lycoris aurea*, bulblets are formed from the axils of the scales on the abaxial surface (Ren et al., 2017), but in *Lycoris radiata* they are preferentially formed on the junctions of the innermost layers of scales and on the basal plate (Xu et al., 2020). During formation, they initially present as axillary buds and gradually develop into bulblets.

In *Lycoris* species and other flowering species with bulbs, including *Lilium* and *Hippeastrum*, bulblet differentiation is mainly determined by carbohydrate metabolism in both bulblet and mother scales. Starch in the mother scale can be degraded into soluble sugars, which are transported to and promote bulblet formation and development through the starch synthesis in bulblets (Zhang et al., 2013; Li et al., 2014; Ren et al., 2017; Xu et al., 2020). In previous studies, sucrose and starch metabolism were found to have a key function in bulblet development into a new bulb, which is controlled by a series of enzymes.

Sucrose can be cleaved by sucrose synthase (SUS; Angeles-Núñez and Tiessen, 2010), and glucose, one of the products, can be used as a direct substrate for starch synthesis under the control of three major starch synthesis-related enzymes: glucose-1-phosphate adenylyltransferase (AGPase), soluble starch synthase (SSS), and granule-bound SS (GBSS; Miao et al., 2016) – AGPase has the dominant promoting effect on the bulblet development process in *Lycoris* (Xu et al., 2020). Previous studies have revealed that AGPase activity could be determined by genes encoding its large and small subunits, as the overexpression of these genes could enhance AGPase activity, resulting in the increase in starch content (Li et al., 2011; Kang et al., 2013). We also proposed that the overexpression of genes encoding AGPase subunits through transgenic and other molecular biology approaches could lead to significant promotion of AGPase activity in *Lycoris* bulbs, which may result in higher proliferation efficiency of the bulblets (Xu et al., 2020). However, because of the lack of a transformation system, it is currently not possible to improve the reproductive efficiency of *Lycoris* through transgenic approaches. Therefore, it is necessary to find another approach that is easy to operate.

In the cultivation of other bulbous ornamental species, exogenous hormones, most commonly auxins and cytokinins (CKs), are widely used for the promotion of bulblet propagation efficiency. During the scale cutting of lily, the application of auxins, including indole-3-butyric acid (IBA) and 1-naphthaleneacetic acid (NAA), can increase the number of bulblets (Wang et al., 2014; Zhang et al., 2014). In *Hyacinthus orientalis*, the application of IBA and 6-benzylaminopurine (BA; a CK) was also found to have a positive effect on bulblet propagation (Sun et al., 2008). However, in *Hippeastrum vittatum* (Amaryllidaceae), the application of exogenous auxin and gibberellic acid (GA) did not result in scale propagation and bulblet development, and the treatment with NAA significantly increased the number of rotten scales (Zhang et al., 2013). Thus, the effect of auxin on the regulation of bulblet development in *Lycoris* should be further examined. In addition, in *Lilium* species, the application of paclobutrazol (PBZ), an inhibitor of GA biosynthesis, was found to have a dose-dependent effect on the bulblets development, with low concentrations promoting and high concentrations inhibiting bulblet development (Wu et al., 2016, 2017). Other studies have also indicated that GA may promote shoot growth and multiplication rather than bulblet development, whereas other hormones and plant growth regulators, such as abscisic acid (ABA), daminozide, and chlorocholine chloride, could significantly improve the average bulblet size (Yadav et al., 2013). However, the effect of these hormones on bulblet development in *Lycoris* species remains unclear.

Understanding the changes in endogenous hormone regulation during the bulblet development process could provide a theoretical basis for the application of exogenous hormones in order to improve the proliferation efficiency of bulblets in production. Previous research on *Lilium* suggested that increasing auxin levels while decreasing CK levels might be useful for promoting bulblet development, whereas increasing only CK levels could be used to promote bulblet formation (Kumar et al., 2007; Tang et al., 2010). Our recent study on the bulblet development process in *L. radiata* indicated that endogenous auxin, CK, GA, brassinosteroids (BR), jasmonic acid (JA), and salicylic acid (SA) may have promoting effects on bulblet formation, whereas ABA and ethylene may have suppressing effects on bulblet formation (Xu et al., 2020), but the effect of these hormones should be further verified by external application to test their suitability for regulating bulblet development in *Lycoris*.

In the present study, we first investigated the effect of different exogenous hormones, including nine kinds of hormones with three application concentrations, on the regulation of bulblet development efficiency in *L. radiata*. Based on these results, we selected four hormones that had a significant promoting or inhibitory effect on bulblet development to investigate the physiological mechanisms that occur during the regulatory process by these four hormones. This included the analysis of changes in endogenous hormone content, carbohydrate content, starch synthesis, metabolic enzyme activity, and the expression patterns of genes related to carbohydrate metabolism. We expect that our results would provide both a theoretical and a practical basis for the regulation of bulblet development efficiency by exogenous hormone application in *L. radiata*, which would be useful for improving bulblet proliferation efficiency in the production of *Lycoris* species or other bulbous ornamentals, such as lilies, tulips, and *Hippeastrum* species.

## Materials and Methods

### Plant Materials and Treatment

*Lycoris radiata* bulbs used in this study were obtained from the *Lycoris* germplasm repository of the Nanjing Botanical Garden Memorial Sun Yat-Sen (NBG) in 2016, transplanted to the experimental base at the Shanghai Academy of Agricultural Sciences (Qingpu District, Shanghai, China), and cultivated for more than 3 years.

At the end of June, when *L. radiata* bulbs were dormant, bulbs with a diameter of 2.9 ± 0.2 cm were selected. After removing the roots and dried scales, the bulbs were cleaned and cut twice across the basal plate (but not completely cut off). After cutting, the bulbs were immediately immersed into solutions of hormones at different concentrations, as shown in Table 1. A total of 40 bulbs were prepared for each treatment, with three replicates per treatment. After 30 min of treatment, the bulbs were placed upwards into square plates and covered with turf soil (PINDSTRUP, Denmark). All plates were then placed in plant growth chambers for bulblet formation and development, under the following conditions: 16 h light/8 h dark photoperiod (8,000 lux), 25/20°C (day/night), and relative humidity of 80%. The experiment started on July 26, 2019, and lasted for almost two and a half months. On October 14, 2019, the bulbs with newly formed bulblets were collected, and the propagation efficiency of the bulblets from different treatments was investigated.

**Table 1:** The design of different hormone treatments in the present study.

Treatments	Hormones	Concentration (mg·L^−1)^	Treatments	Hormones	Concentration (mg·L^−1)^
T1(Control)	Distilled water	–	T6-2	ABA	50
T2-1	NAA	5	T6-3	ABA	100
T2-2	NAA	10	T7-1	BR	1
T2-3	NAA	20	T7-2	BR	2
T3-1	6-BA	25	T7-3	BR	4
T3-2	6-BA	50	T8-1	JA	25
T3-3	6-BA	100	T8-2	JA	50
T4-1	GA_3_	25	T8-3	JA	100
T4-2	GA_3_	50	T9-1	SA	50
T4-3	GA_3_	100	T9-2	SA	100
T5-1	PBZ	50	T9-3	SA	200
T5-2	PBZ	100	T10-1	Ethrel	50
T5-3	PBZ	200	T10-2	Ethrel	100
T6-1	ABA	25	T10-3	Ethrel	200

*Abbreviations: NAA: 1-naphthylacetic acid; 6-BA: 6-benzylamino-purine; GA_3_: gibberellic acid; PBZ: paclobutrazol; ABA: abscisic acid; BR: brassinosteroid; JA: jasmonic acid; SA: salicylic acid*.

Based on our previous observation, we found that exogenous GA_3_ could significantly inhibit bulblet development in *L. radiata*, whereas PBZ, ABA, and ethrel could promote it. Thus, we used another group of *L. radiata* bulbs to determine the changes in physiological indices among these four hormones related to the regulation of bulblet development. On October 21, 2019, 400 bulbs of the same diameter were selected after pretreatment and cut as described above. These bulbs were divided into five groups and treated with different hormones: bulbs in the GA_3_ treatment were treated with 50 mg L^−1^ GA_3_, bulbs in the PBZ treatment were treated with 200 mg L^−1^ PBZ, bulbs in the ABA treatment were treated with 50 mg L^−1^ ABA, and bulbs in the ethrel treatment were treated with 50 mg L^−1^ ethrel. The control group was treated with the same volume of distilled water, and it was designated as a control treatment. The bulbs were placed in plates filled with turf soil, and the plates were placed in plant growth chambers under the same conditions as described above.

### Sampling

At 0, 3, 7, 14, and 30 days after treatment (DAT), 10 bulbs were randomly selected from each treatment. Sampling was performed in triplicate with each replicate consisting of three or four bulbs. Samples of tissues surrounding the zones of bulblet emergence were collected as described previously (Xu et al., 2020), and these samples were considered as bulblet samples. The remaining tissues were also collected and considered as mother-scale samples. All materials were frozen in liquid nitrogen for 30 min and stored at −80 until further analyses.

### Determination of Endogenous Hormone Content

Four types of hormones, including auxin, CK, GA, and ABA, were detected by MetWare^1^ using the AB Sciex QTRAP 6500 LC-MS/MS platform according to the methods described previously (Floková et al., 2014; Cui et al., 2015; Šimura et al., 2018; Xiao et al., 2018).

Approximately 50 mg of each plant material was frozen and ground into powder in liquid nitrogen, after which it was extracted with methanol/water/formic acid (15:4:1, V/V/V). The combined extracts were evaporated until dry under a nitrogen gas stream, reconstituted in 80% methanol (V/V), and filtered (PTFE; 0.22 μm; Anpel) before LC-MS/MS analysis.

The sample extracts were then analyzed using an LC-ESI-MS/MS system (HPLC, Shim-pack UFLC SHIMADZU CBM30A system^2^; MS, Applied Biosystems 6500 Triple Quadrupole^3^). The analytical conditions were as follows: HPLC: column, Waters ACQUITY UPLC HSS T3 C18 (1.8 μm, 2.1 mm × 100 mm); solvent system, water (0.05% acetic acid): acetonitrile (0.05% acetic acid); gradient program, 95:5 V/V (water: acetonitrile, the same below) at 0 min, 95:5 V/V at 1 min, 5:95 V/V at 8 min, 5:95 V/V at 9 min, 95:5 V/V at 9.1 min, 95:5 V/V at 12 min; flow rate, 0.35 ml/min; temperature, 40°C; injection volume, 2 μl. The effluent was alternatively connected to an ESI-triple quadrupole-linear ion trap (QTRAP)-MS.

AB 6500 QTRAP LC/MS/MS system was equipped with an ESI Turbo Ion-Spray interface, operating in both positive and negative ion modes and controlled by Analyst 1.6 software (AB Sciex). The ESI source operation parameters were as follows: ion source, turbo spray; source temperature, 500°C; ion spray voltage (IS), 4,500 V; curtain gas (CUR), 35.0 psi; collision-activated dissociation (CAD), medium. The declustering potential (DP) and collision energy (CE) for individual MRM transitions were performed with further DP and CE optimization. A specific set of MRM transitions was monitored for each period according to the plant hormones eluted within this period.

### Determination of Carbohydrate Content

The contents of starch, total soluble sugars, and sucrose were measured as previously reported (Miao et al., 2016). Approximately 0.5 g of powdered samples ground in liquid nitrogen were incubated with 4 ml of 80% ethanol at 80°C for 30 min, after which they were centrifuged at 8,000 g for 20 min. After decolorization with activated carbon, the supernatant was used for the measurement of soluble sugars, including sucrose. The precipitates were successively suspended in 9.2 M and 4.6 M HClO_4_ to extract starch. Total soluble sugar and starch concentrations were then determined using the anthrone reaction, and the sucrose content was measured by hydrolysis and the resorcinol reaction.

### Determination of the Activities of Enzymes Involved in Sucrose Metabolism and Starch Synthesis

The activities of sucrose metabolism enzyme (SUS) and three major starch synthesis enzymes (AGPase, SSS, and GBSS) were determined according to the method described previously (Nakamura et al., 1989; Wu et al., 2017). All procedures were conducted at a temperature of 4°C. Approximately 0.5 g of each sample was ground in liquid nitrogen and extracted with a buffer solution (5 ml g^−1^ sample fresh weight, FW) containing 100 mM of 4-(2-hydroxyethyl)-1-piperazineethanesulfonic acid (HEPES)-NaOH frozen extraction buffer (pH 7.5), 8 mM of MgCl_2_, 2 mM of ethylenediaminetetraacetic acid (EDTA), 50 mM of 2-mercaptoethanol, 12.5% of glycerol, and 1% (0.01 g/ml) of insoluble polyvinylpyrrolidone-40. After centrifuging at 30,000 g for 30 min, the supernatant was used for the determination of SUS, AGPase, and SSS activities, whereas the sediment was used for the determination of GBSS activity. The enzyme activities were compared with the soluble protein content, which was determined by a modified Bradford method (Bradford, 1976).

### RNA Extraction, cDNA Synthesis, and qRT-PCR Assays

Total RNA extraction and cDNA synthesis were carried out using a MiniBEST Plant RNA Extraction Kit and PrimeScript™ RT Reagent Kit with gDNAEraser Kit (TaKaRa, Dalian, China), respectively, following the manufacturer’s protocol. RNA quantity and quality were measured using a NanoDrop 2000 Spectrophotometer (Thermo Fisher Scientific, United States), and RNA samples with the absorption ratios of *A*_260/280_ = 1.8–2.2 were selected to synthesize cDNA. For qRT-PCR, the obtained cDNA was diluted 10-fold using nuclease-free water.

qRT-PCRs were run on a Roche LightCycler 480II System using SYBR Premix Ex Taq™ (TaKaRa, Dalian, China). The relative expression levels of target genes were calculated according to a method described previously (Xu et al., 2015). *Actin* was used as the reference gene. Three biological replicates were included per treatment. The gene accession numbers and primers used for qRT-PCR are listed in Supplementary Table S1, based on our full-length transcriptome database of *L. radiata* (unpublished data).

### Statistical Analyses

All statistical analyses were conducted using SPSS 16.0. The means of values were compared by standard analysis of variance followed by least significant difference tests, and *p* < 0.05 was considered significant.

## Results

### The Effects of Different Concentrations and Different Types of Exogenous Hormones on Bulblet Development Efficiency in *Lycoris radiata*

After the application of different exogenous hormones, bulblet development efficiency of *L. radiata* showed significant differences among several treatments (Table 2). Compared to the control treatment (T1), the application of exogenous GA_3_ (T4 treatments) significantly inhibited the propagation coefficient and weight of bulbs, and this inhibitory effect was enhanced with the increase in GA_3_ concentration. In contrast, we found that the application of PBZ, a GA biosynthesis inhibitor, significantly promoted the number and weight of newly formed bulblets, especially in the T5-3 treatment (200 mg·L^−1^ PBZ). Interestingly, we found that ethrel application also promoted the propagation coefficient and weight of bulbs (T10-3 treatment; 200 mg·L^−1^ ethrel), whereas ABA application promoted the weight of newly formed bulblets but had no significant effect on their number (T6-2 treatment; 50 mg·L^−1^ ABA). In addition to PBZ, ABA, and ethrel application, we found that the application of 10 mg·L^−1^ NAA and 25 mg·L^−1^ 6-BA promoted the average proliferation numbers and weight of surviving bulbs (T2-2 and T3-1 treatments) even more than the treatments with PBZ, ABA, and ethrel. However, compared to that after PBZ, ABA, and ethrel addition, NAA and 6-BA can cause an increase in the rotting rate of bulbs, resulting in an indistinctive increase in the propagation coefficient of the bulbs compared to that in the control treatment. Thus, we concluded that the application of exogenous NAA and 6-BA was not suitable for use during the reproduction process of *L. radiata*, but the applications of PBZ, ABA, and ethrel are suitable and could be fully utilized. Similar to NAA and 6-BA, the application of SA (T9 treatments) also increased the rotting rate of bulbs, which was enhanced by the increase in SA concentration. In addition, the application of BR and JA had no significant effect on the propagation coefficient of bulbs. Although the application of 50 mg·L^−1^ JA (T8-2 treatment) slightly increased the propagation coefficient of bulbs, the propagation weight was not significantly increased.

**Table 2:** The effect of different hormones on the bulblet development efficiency in *L. radiata*.

Numbers	Treatments	Survival rate (%)	Average proliferation numbers	Propagation coefficient of bulbs	Average proliferation weight	Propagation weight of bulbs
1	T1	97.5ab	5.18efghi	5.05efg	4.78fghi	4.66efghi
2	T2-1	100a	5.05ghi	5.05efg	4.94efgh	4.94cdef
3	T2-2	82.5d	6.13a	5.08efg	5.4cd	4.46ghijk
4	T2-3	92.5bc	5.18efghi	4.79g	4.51ijk	4.16jkl
5	T3-1	85d	6.09a	5.17def	5.81b	4.93cdef
6	T3-2	87.5cd	5.49bcdefg	4.79g	5.04defg	4.42ijk
7	T3-3	85d	5.19efghi	4.41h	4.62hij	3.96l
8	T4-1	95ab	3.94j	3.75i	2.01m	1.88n
9	T4-2	95ab	3.07k	2.92j	1.35n	1.32o
10	T4-3	82.5d	2.77k	2.29k	1.09n	0.9p
11	T5-1	100a	5.8abc	5.79a	5.31cde	5.3bc
12	T5-2	97.5ab	5.77abcd	5.64abc	5.56bc	5.41b
13	T5-3	97.5ab	5.9ab	5.76ab	6.39a	6.25a
14	T6-1	97.5ab	5.47bcdefgh	5.34cde	5.18def	5.06cde
15	T6-2	97.5ab	5.31cdefghi	5.18def	5.64bc	5.50b
16	T6-3	95ab	5.24defghi	4.99efg	4.65ghij	4.44hijk
17	T7-1	100a	4.93hi	4.93fg	4.16kl	4.16jkl
18	T7-2	97.5ab	4.86i	4.76g	4.29jkl	4.2jkl
19	T7-3	100a	4.84i	4.84fg	4.11l	4.11kl
20	T8-1	92.5bc	5.6abcdefg	5.17def	4.64hij	4.29ijkl
21	T8-2	100a	5.64abcdef	5.64abc	4.91fgh	4.91cdef
22	T8-3	100a	5.1fghi	5.1defg	4.55hij	4.55fghij
23	T9-1	100a	5.44bcdefgh	5.43bcd	4.85fghi	4.85defgh
24	T9-2	92.5bc	5.16efghi	4.77g	4.85fghi	4.48ghijk
25	T9-3	75e	5.18efghi	3.88i	4.04l	3.03m
26	T10-1	100a	5.55bcdefg	5.55abc	5.13def	5.13cd
27	T10-2	100a	5.67abcde	5.67abc	4.86fghi	4.86defg
28	T10-3	100a	5.85abc	5.85a	5.57bc	5.57b

*Treatments were the same as those in Table* *1**. Average proliferation numbers and weights were calculated based on the number of surviving bulbs, whereas the propagation coefficient and weight were calculated based on the number of all bulbs in each treatment. The values in each column followed by different letters indicate significant difference at p = 0.05 (n = 40)*.

### Morphological Description of the Bulblet Development Process in *Lycoris radiata* Treated With Different Exogenous Hormones

**Figure 1:**
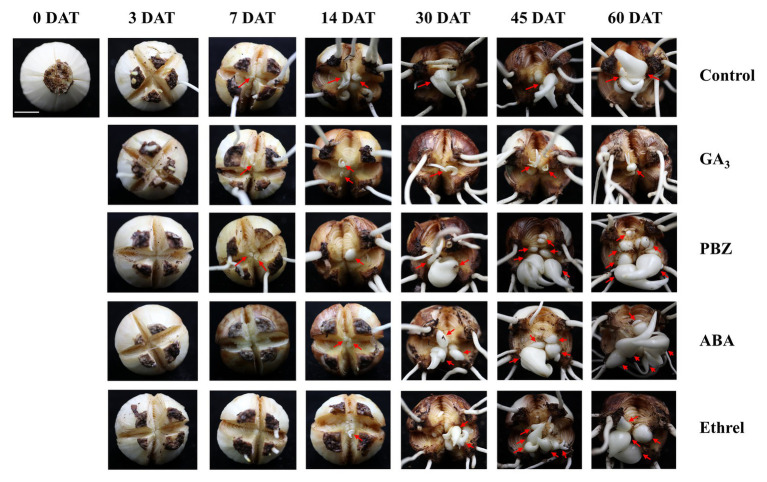
Morphological characteristics of bulblet development under different hormone treatments of *Lycoris radiata*. The arrows indicate the position where the axillary bud is formed and the bulblet develops. Pictures were taken at 0, 3, 7, 14, 30, 45, and 60 days after treatment (DAT). Bar = 1 cm.

In order to study the dynamic changes in the bulblet development process under GA_3_, PBZ, ABA, and ethrel treatments in *L. radiata*, we observed the formation and development of bulblets at 0, 3, 7, 14, 30, 45, and 60 DAT (Figure 1). Consistent with our previous observation, bulblets in the control treatment initially presented as axillary buds at 7 DAT, and gradually developed into bulblets. GA_3_ and PBZ treatments showed no significant difference during the axillary bud formation process at 7 DAT, but there was a distinct difference in bulblet development from 14 DAT. Compared to the control treatment, the GA_3_ treatment significantly inhibited bulblet development, and axillary buds could hardly elongate and develop. In contrast, bulblet development in the PBZ treatment was quicker than in the control and GA_3_ treatments, along with higher quantities and qualities of bulblets, indicating that the PBZ treatment could promote bulblet development in *L. radiata*. In addition, we found that ABA and ethrel treatments can delay the bulblet formation process, as axillary bud formation started up to 14 DAT. Interestingly, the processes of bulblet development in the ABA and ethrel treatments were accelerated from 30 DAT, resulting in the formation of more bulblets than in the control and GA_3_ treatments.

### Changes in Endogenous Hormone Contents During Bulblet Development in *Lycoris radiata* Under Different Exogenous Hormone Treatments

We measured the changes in the contents of four major types of endogenous hormones, and we successfully detected nine hormones, including indole-3-acetic acid (IAA), methyl indole-3-acetate (MeIAA), Indole-3-carboxaldehyde (ICAld), and N6-Isopentenyladenine (IP, one type of endogenous CK), four types of GA (GA_3_, GA_15_, GA_19_, GA_24_), and ABA, during bulblet development under different exogenous hormone treatments.

**Figure 2:**
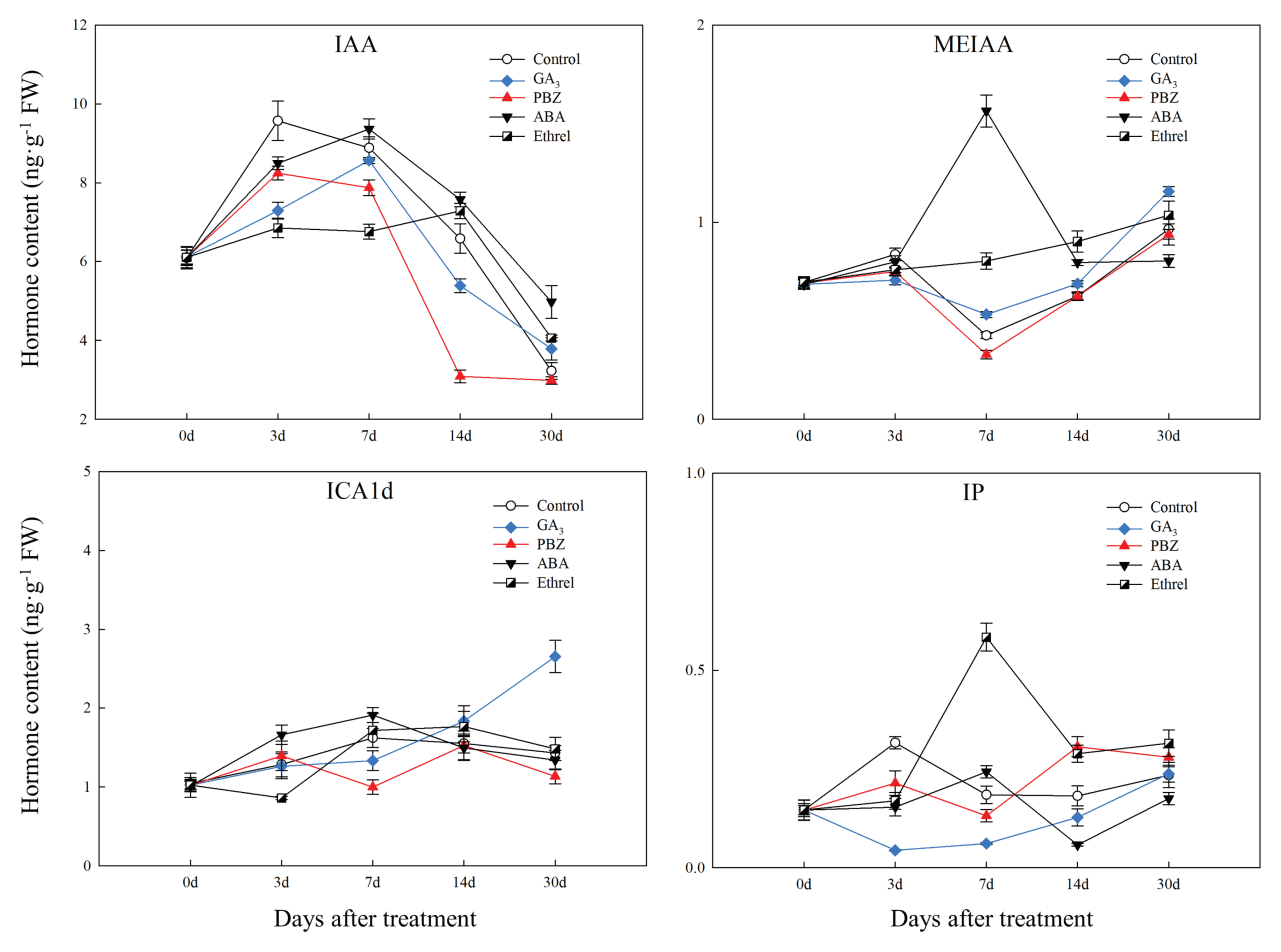
Changes in three types of endogenous auxin and IP contents during the bulblet development process of *L. radiata* under different hormone treatments. IAA: indole-3-acetic acid; MeIAA: methyl indole-3-acetate; ICAld: Indole-3-carboxaldehyde; IP: N6-isopentenyladenine, a type of endogenous cytokinin (CK).

The IAA content in all treatments first increased and then decreased. From 7 DAT, IAA content in the ABA-treated bulblets was much higher than that in bulblets treated with other agents (Figure 2). MeIAA content in the present study was increased in all treatments from 7 DAT, except in the ABA treatment, which was the highest at 7 DAT (Figure 2). In addition, our results showed no significant changes in ICAld contents in all treatments from 0 to 14 DAT, until an increase occurred in the GA_3_ treatment at 30 DAT (Figure 2).

**Figure 3:**
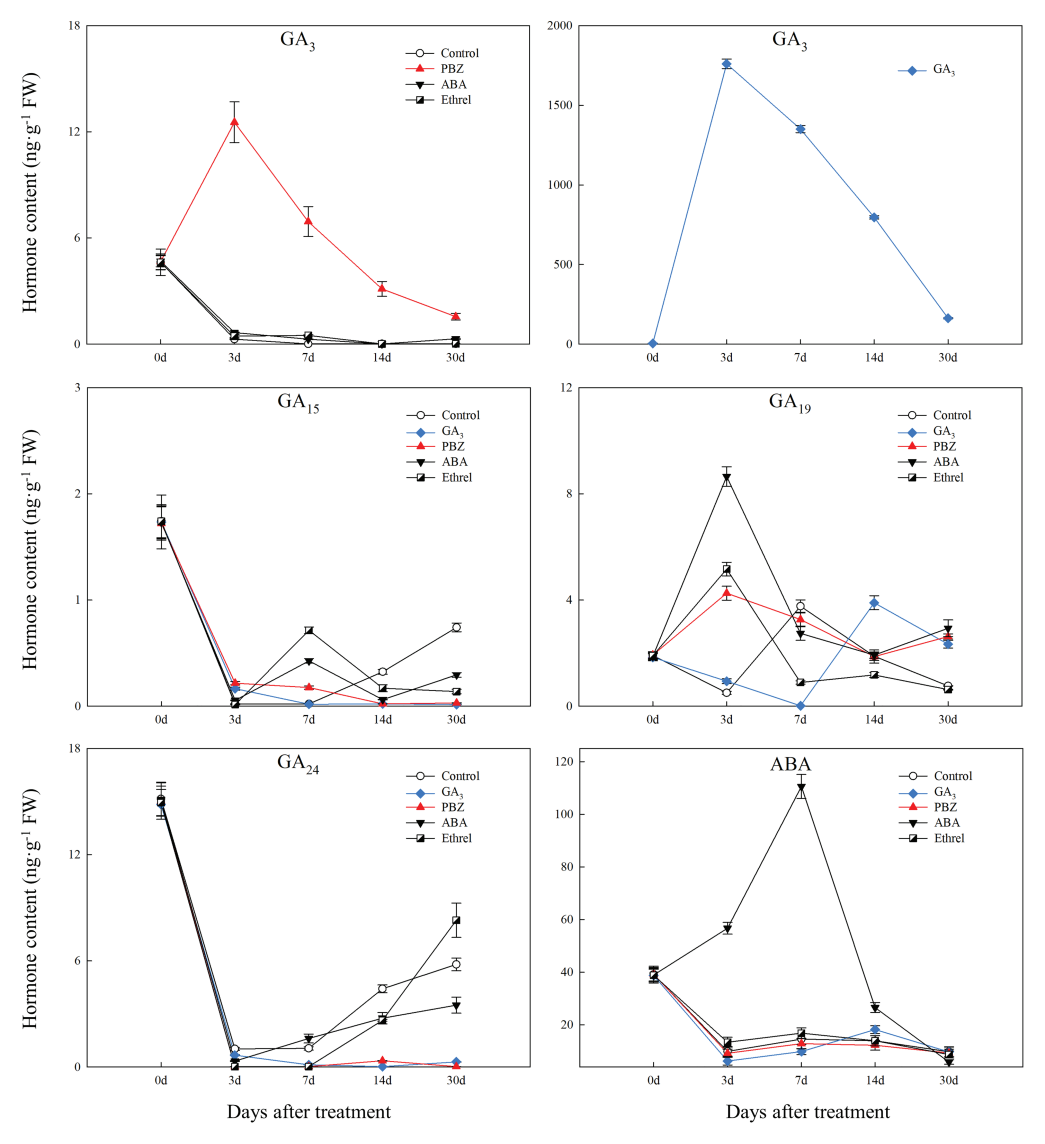
Changes in four types of endogenous gibberellic acid (GA) and abscisic acid (ABA) contents during the bulblet development process of *L. radiata* under different hormone treatments.

Regarding endogenous CK contents, we only successfully detected the content of IP in the present study. We found that IP content was significantly decreased in the GA_3_ treatment from 0 to 7 DAT, whereas in the PBZ, ABA, and ethrel treatments, it increased from 7 DAT, especially in the ethrel treatment (Figure 2). In addition, the changes in ABA content were quite similar among control, GA_3_, PBZ, and ethrel treatments showing a decrease during the bulblet development process (Figure 3). Furthermore, we found that ABA content in the ABA treatment was significantly increased from 0 to 7 DAT, which may be a consequence of the application of exogenous ABA (Figure 3). Interestingly, ABA content in this treatment significantly decreased from 7 DAT, reaching a nadir at 30 DAT (Figure 3).

We successfully detected four types of GAs. Among them, GA_3_ content in the control, ABA, and ethrel treatments decreased during the bulblet development process, whereas that in the GA_3_ and PBZ treatments first increased from 0 to 7 DAT, and then decreased (Figure 3). Regarding other GAs, the changes in GA_15_ and GA_24_ contents showed some similarities: they both significantly decreased from 0 to 3 DAT, but subsequently increased in the control, ABA, and ethrel treatments; however, their contents in the GA_3_ and PBZ treatments were very close to zero from 3 to 30 DAT (Figure 3).

### Changes in Carbohydrate Contents During Bulblet Development in *Lycoris radiata* Under Different Exogenous Hormone Treatments

**Figure 4:**
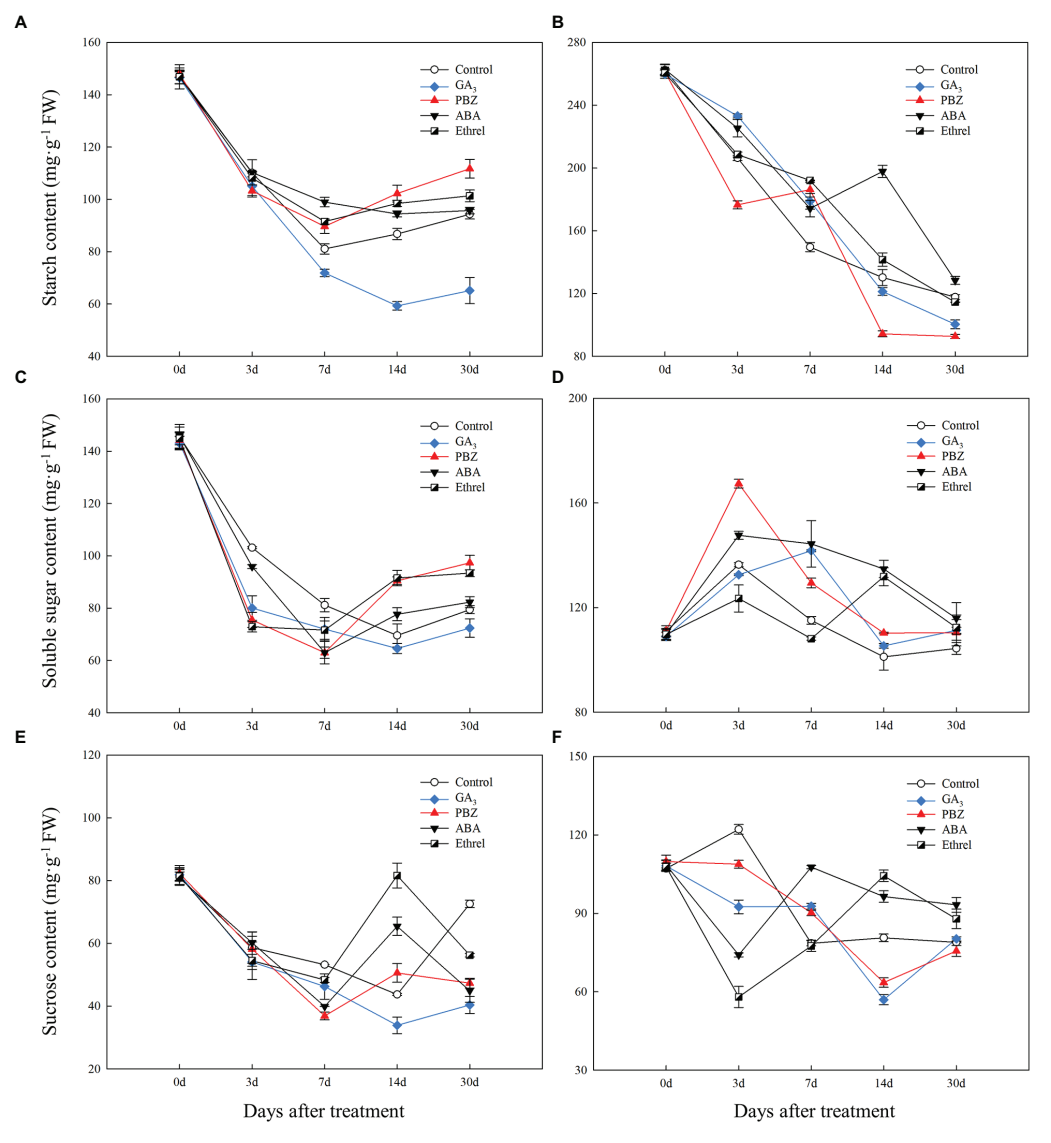
Changes in carbohydrate contents during the bulblet development process of *L. radiata* under different hormone treatments. **(A,B)** starch; **(C,D)** soluble sugar; **(E,F)** sucrose; **(A,C,E)** bulblets and zones where bulblets are formed; **(B,D,F)** mother scales.

During bulblet formation and development in the control treatment, starch content decreased at first, reaching a nadir at 7 DAT, and then increased (Figure 4A). Starch contents in PBZ, ABA, and ethrel treatments (particularly in PBZ treatment) were higher than in control treatment from 7 DAT; however, starch content in GA_3_ treatment continuously decreased from 0 to 14 DAT, and it was significantly lower than in other treatments (Figure 4A). Soluble sugar and sucrose contents had similar changing trends as starch, i.e., they decreased at first and then increased (Figures 4C,E). Interestingly, we found that the decrease rates of soluble sugar contents in the GA_3_, PBZ, and ethrel treatments were much faster than that in the control treatment from 0 to 3 DAT, whereas their increase rates were also faster from 14 DAT, except for those in the GA_3_ treatment (Figure 4C). The sucrose content showed the most significant differences among all treatments at 14 DAT; it was the highest in the ethrel treatment, and the lowest in the GA_3_ treatment (Figure 4E).

In our previous study, we proposed that the starch in the mother scales could be degraded into soluble sugars, transported to bulblets, and could promote bulblet formation and development (Xu et al., 2020). Consistent with this, we found that starch content in the mother scales decreased during the bulblet development process under different treatments (Figure 4B). In addition, the decrease rate of starch content was the fastest in the PBZ treatment, followed by that in the GA_3_ treatment (Figure 4B). Interestingly, we found that the soluble sugar contents in the mother scales was increased at 3 DAT in all treatments, and that it was the highest in the PBZ treatment (Figure 4D). After 3 DAT, the soluble sugar contents started to decrease, and were lower in the control, GA_3_, and PBZ treatments than those in the ABA, and ethrel treatments (Figure 4D). The sucrose content in the control treatment slightly increased and then decreased. In addition, we found that the changes in sucrose contents in the GA_3_ and PBZ treatments were different from those in the ABA and ethrel treatments, showing the fastest decrease rates at 14 and 3 DAT, respectively (Figure 4F).

### Changes in Sucrose Metabolism and Starch Synthesis Enzymes During Bulblet Development in *Lycoris radiata* Under Different Exogenous Hormone Treatments

We measured the changes in the activity of SUS and three starch synthesis enzymes (AGPase, SSS, and GBSS) during the bulblet development process under different hormone treatments.

**Figure 5:**
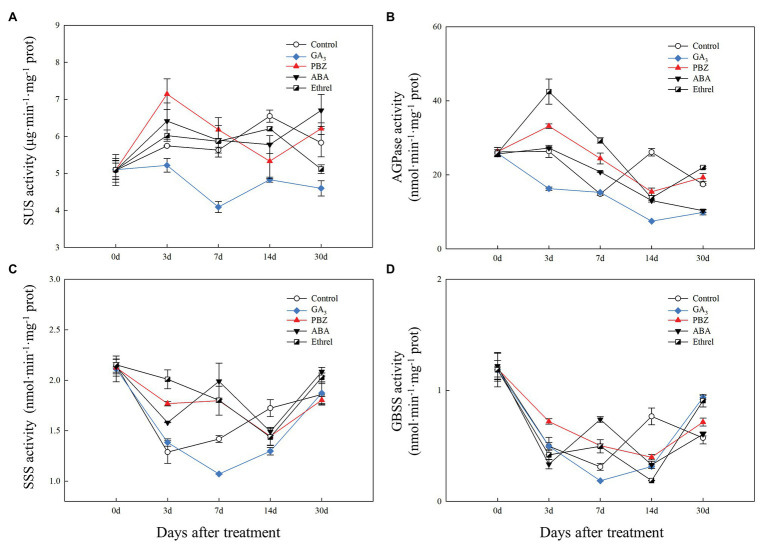
Changes in the activities of sucrose metabolism and starch synthesis enzymes during the bulblet development process of *L. radiata* under different hormone treatments. **(A)** SUS: sucrose synthase enzyme; **(B)** AGPase: glucose-1-phosphate adenylyltransferase **(C)** SSS: soluble starch synthase sucrose; **(D)** GBSS: granule-bound starch synthase.

Compared to that in the other treatments, SUS activity in the GA_3_ treatment remained at a relatively low level during the entire bulblet development process, whereas its activity in other treatments increased from 0 to 3 DAT, and then stayed at a relatively higher level than that in the GA_3_ treatment. Among the other treatments, SUS activity at 3 DAT was highest in the PBZ treatment, followed by the ABA, ethrel, and control treatments (Figure 5A).

Regarding starch synthesis enzymes, the changes in AGPase activity showed some similarities to the changes in SUS activity, and the activity was the highest from 3 to 7 DAT in the ethrel treatment, followed by PBZ and ABA treatments (Figure 5B). Consistent with SUS activity, AGPase activity in the GA_3_ treatment was the lowest among all treatments during all time periods (Figure 5B). However, it was interesting that the activities of two other starch synthesis enzymes, SSS and GBSS, in GA_3_ treatment first decreased and then increased from 7 DAT, in contrast with other treatments (Figures 5C,D). Interestingly, the activities of SSS and GBSS showed the same patterns at 7 DAT; they were the highest in the ABA treatment, followed by the PBZ, ethrel, control, and GA_3_ treatments in that order (Figures 5C,D).

### Changes in the Expression Levels of Genes Involved in Sucrose Metabolism and Starch Synthesis During Bulblet Development in *Lycoris radiata* Under Different Exogenous Hormone Treatments

**Figure 6:**
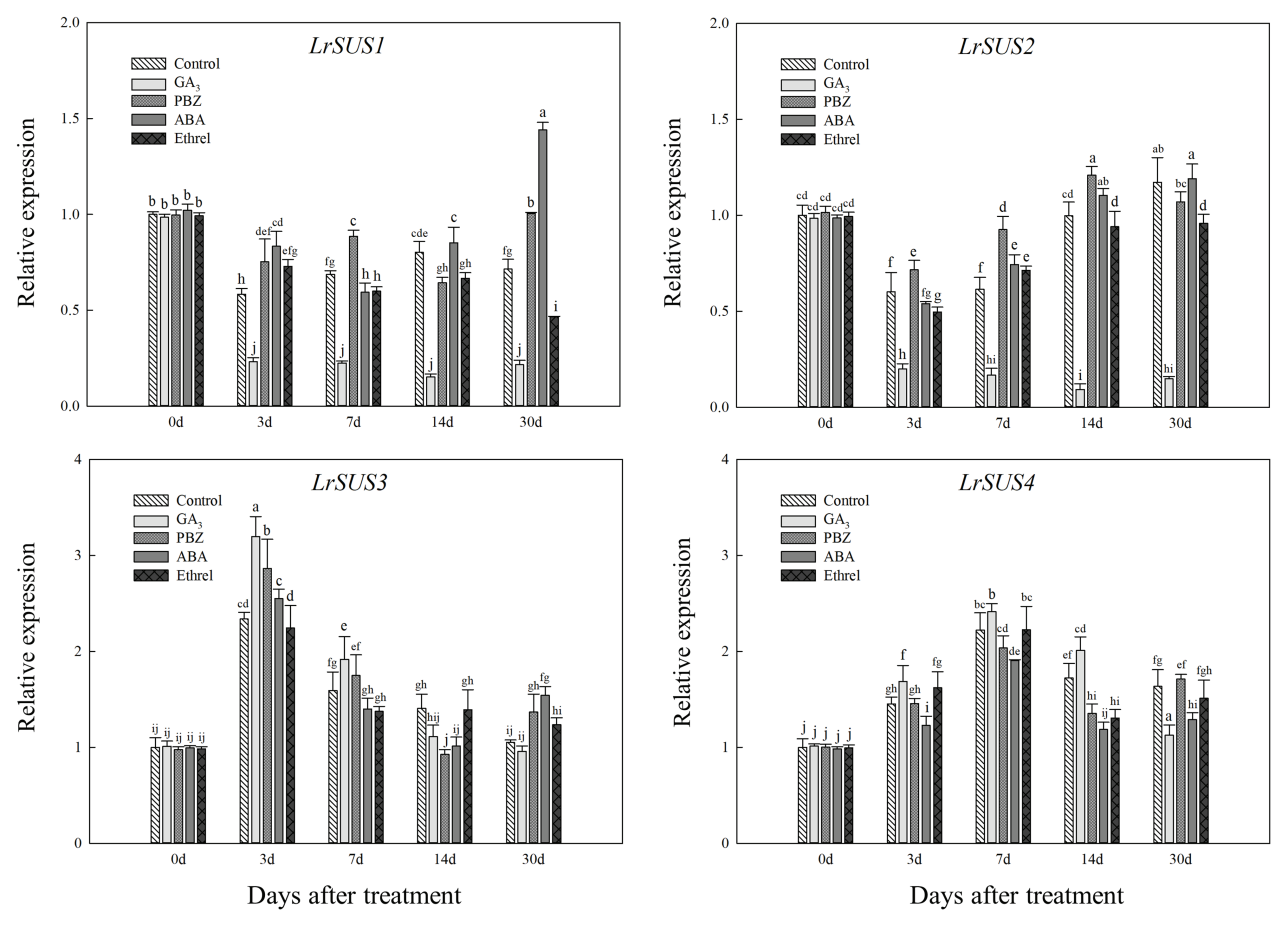
Changes in the expression levels of *LrSuS1*, *LrSuS2*, *LrSuS3*, and *LrSuS4* during the bulblet development process of *L. radiata* under different hormone treatments. For qRT-PCR analysis, the values obtained for the control samples at 0 DAT were arbitrarily set at 1.0.

Based on our full-length transcriptome database of *L. radiata*, we selected several genes encoding carbohydrate metabolism enzymes to detect their expression patterns under different treatments. As shown in Figure 6, we observed differences in the expression levels of four genes encoding the SUS enzyme, and only the changes in the expression of *LrSUS1* and *LrSUS2* genes were consistent with the changes in SUS activity. The expression levels of these two genes in the GA_3_ treatment were significantly decreased during the whole treatment process (Figure 6). In addition, the expression patterns of *LrSUS3* and *LrSUS4* genes were opposite of those of *LrSUS1* and *LrSUS2* genes, and thus, the changes in SUS activity under different treatments were opposite, as well, which may be a consequence of the feedback regulation of these genes.

**Figure 7:**
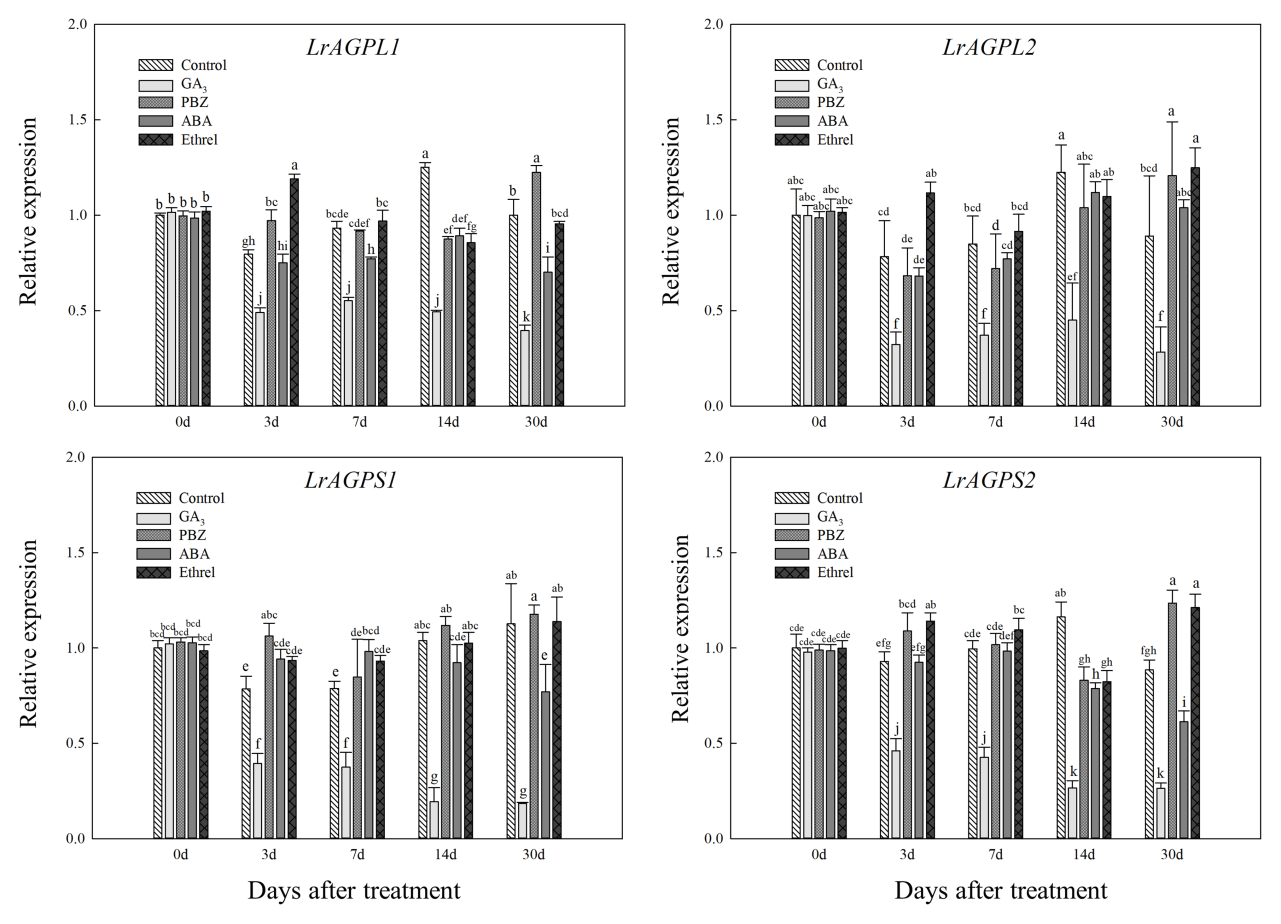
Changes in the expression levels of *LrAGPL1*, *LrAGPL2*, *LrAGPS1*, and *LrAGPS2* during the bulblet development process of *L. radiata* under different hormone treatments. Details are as described in the legend of Figure 6.

Glucose-1-phosphate adenylyltransferase, an enzyme that is critical for starch synthesis and that catalyzes the first step of starch synthesis (Nakkanong et al., 2012), was proved to be composed of two subunits (Morell et al., 1987). Consistent with *LrSUS1* and *LrSUS2* genes, the expression levels of genes encoding the large and small subunits of AGPase were significantly decreased in the GA_3_ treatment, but in the other treatments (mostly PBZ and ethrel), they were increased at several time points, particularly *LrAGPL1*, *LrAGPS1*, and *LrAGPS2* genes at 3 DAT, and *LrAGPL2* and *LrAGPS2* genes at 30 DAT (Figure 7).

**Figure 8:**
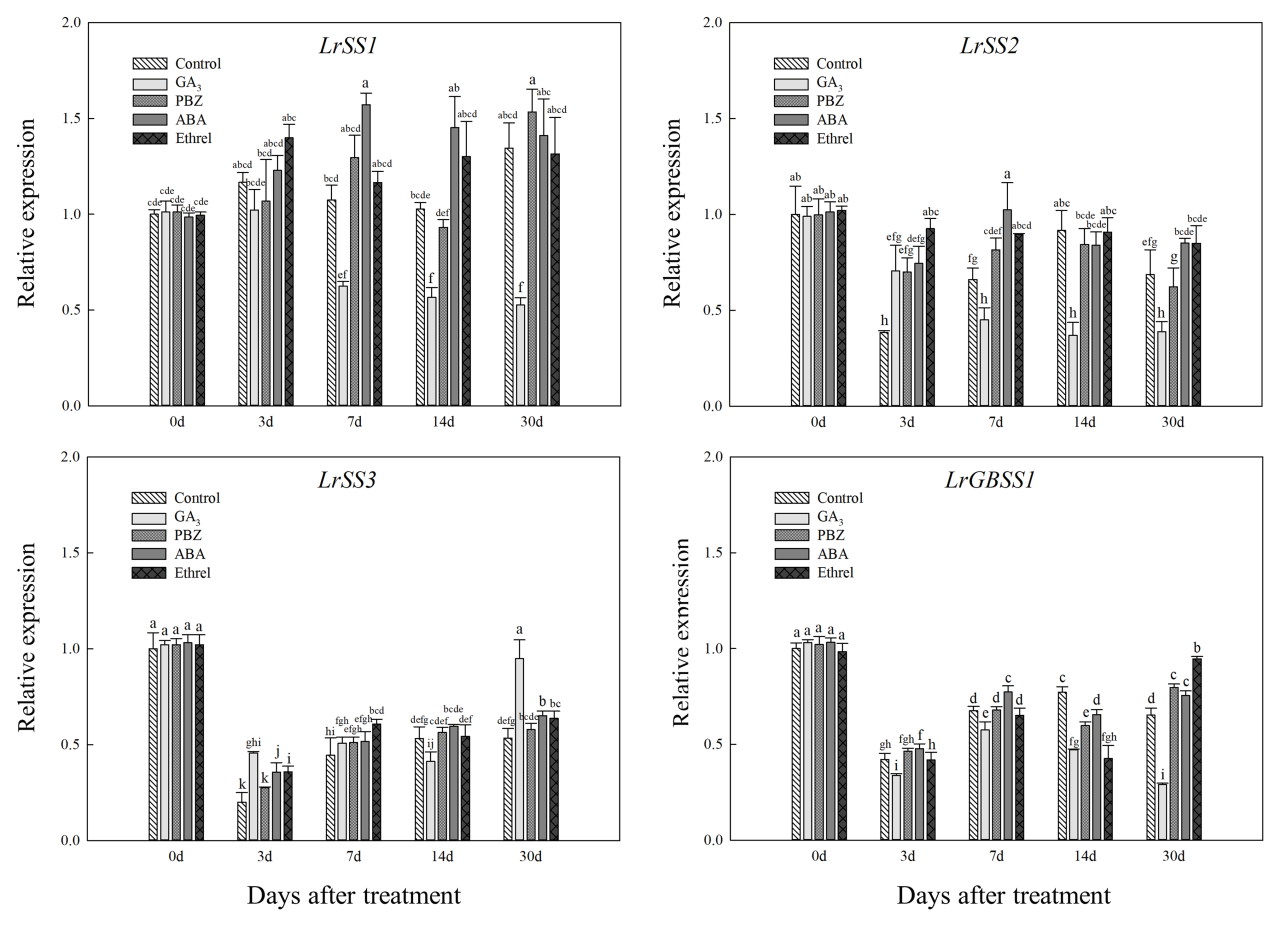
Changes in the expression levels of *LrSS1*, *LrSS2*, *LrSS2*, and *LrGBSS1* during the bulblet development process of *L. radiata* under different hormone treatments. Details are as described in the legend of Figure 6.

In addition, the expression levels of *LrSS1* and *LrSS2* genes encoding the SSS enzyme also showed a decrease in the GA_3_ treatment from 7 to 30 DAT (Figure 8); however, interestingly, the expression level of the *LrSS3* gene in the GA_3_ treatment was first decreased at 14 DAT, after which it significantly increased at 30 DAT, which may be associated with the increase in SSS activity at 30 DAT in this treatment (Figure 8). Moreover, we found that the expression levels of *LrSS1* and *LrSS2* genes in the ABA treatment were higher than those in other treatments, especially for *LrSS1* at 7 DAT (Figure 8), which was also consistent with the changes in SSS activity. The expression levels of the *LrGBSS1* gene encoding the GBSS enzyme showed no significant changes among all treatments, except for the GA_3_ treatment (Figure 8). Unlike the *LrSS3* gene, the expression level of the *LrGBSS1* gene in the GA_3_ treatment at 30 DAT was significantly decreased (Figure 8), which was opposite to the increasing trend of GBSS activity in the GA_3_ treatment.

## Discussion

The slow reproduction efficiency of *Lycoris* species in nature greatly restricts the artificial propagation of their bulbs, and the application of exogenous hormones during practical production processes is an effective way to promote *Lycoris* propagation, i.e., bulblet development efficiency. However, unlike in *Lilium* species, the hormone that could be conducive to bulblet development in *Lycoris* has not been fully revealed to date. In the present study, we first tested the effect of nine different types of hormones on bulblet development in *L. radiata*. We found that exogenous GA significantly inhibited bulblet development in a dose-dependent manner (Table 2; Figure 1). However, we also found that the application of the GA biosynthesis inhibitor PBZ could promote the bulblet development of *L. radiata* (Table 2; Figure 1). In tuberous plants, GA has been shown to have an inhibitory effect on tuber induction (Roumeliotis et al., 2012). Exogenous GA_3_ was found to inhibit tuber formation of potato (*Solanum tuberosum* L.) *in vitro*, and with an increase in exogenous GA_3_ concentration, the tuber volume and tuber number per stolon decreased (Cheng et al., 2018). These results were supported by the results of the present study, which implied that compared to control, GA and PBZ could inhibit and promote bulblet development in *L. radiata*, respectively.

In addition, we found that compared to control, the application of ABA and ethrel could also promote bulblet development, even though their promoting effect was inferior to that of PBZ (Table 2; Figure 1). Previous studies in potatoes showed that exogenous GA_4/7_ promoted stolon elongation and inhibited tuber formation, whereas exogenous ABA stimulated tuberization and reduced stolon length (Xu et al., 1998). Consistently, ABA has been shown to be a positive modulator of potato tuberization, whereas GA was found to be an inhibitor of the process (García et al., 2014), which was similar to the results of the present study. Ethylene has been shown to have an inhibitory effect on tuberization in potato (Mingo-Castel and Kumamoto, 1976), whereas it had the opposite effect on bulblet development in the present study. On the other hand, ethylene has been shown to be involved in different types of plant stress, including that of wound response (Bleecker and Kende, 2000; Ciardi and Klee, 2001), implying that ethrel treatment was beneficial to wound healing of *L. radiata* bulbs after cutting, contributing to subsequent bulblet development.

Bulblet development could partly be determined by the interaction of endogenous plant hormones. Among them, CK is considered to promote bulblet development in *L. radiata* (Xu et al., 2020); although exogenous CK application caused an increase in the rate of rotten bulbs, the average proliferation numbers and weight of surviving bulbs with 25 mg·L^−1^ 6-BA treatment were higher than those of the control (Table 2). Exogenous GA significantly inhibited IP (one type of CK) content from 0 to 7 DAT, whereas PBZ and ethrel could promote it after 7 DAT (Figure 2). GA and CK have been shown to have antagonistic effects on various developmental and molecular processes during plant growth, and GA could inhibit CK response and signaling (Greenboim-Wainberg et al., 2005; Fleishon et al., 2011), which was consistent with our results. In addition, ethrel application significantly promoted IP content from 7 DAT (Figure 2). Previous studies showed that CK concentration and signaling is regulated by the kinase activity of the ethylene receptor ETR1 (Liu et al., 2017), the activity of which could be inhibited by ethylene (Kamiyoshihara et al., 2012). A decrease in ETR1 receptor activity should reduce the activity of CK oxidase and result in increased CK concentration (Liu et al., 2010, 2017). These results imply that the improvement in bulblet development for ethylene could be partly dependent on the promotion of endogenous CK content. However, unlike PBZ and ethrel application, ABA application strongly inhibited IP content from 14 DAT (Figure 2), suggesting that the promotion of bulblet development by ABA is not due to the increase in CK content. In previous studies, ABA was proven to suppress both the content and signaling of CK in plants (Vaseva et al., 2008; Nishiyama et al., 2011; Takatsuka and Umeda, 2019), which agreed with our observation.

In the present study, auxin was also beneficial for the initiation of bulblet formation, as IAA content increased during the early stages of bulblet development and subsequently decreased with bulblet development (Figure 2). Same results were observed during the potato tuberization process (Roumeliotis et al., 2012). Compared to other treatments, exogenous ABA treatment promoted both IAA and MeIAA contents, especially MeIAA content at 7 DAT (Figure 2). MeIAA is an inactive form of IAA, and the activity of MeIAA *in vivo* is a consequence of the action of free IAA, which is generated from MeIAA upon hydrolysis by one or more plant esterases (Qin et al., 2005; Yang et al., 2008). Auxin tends to act downstream of ABA in the regulation of many processes, including light-grown roots, seed germination, and cotyledon expansion (Emenecker and Strader, 2020). In regulating root growth, ABA plays positive roles in regulating root system growth by modulating MAPK and auxin signaling and the cell cycle (Zhao et al., 2015). Similarly, another study found that ABA could promote auxin biosynthesis and polar auxin transport, leading to auxin accumulation in the long root hair zone (Wang et al., 2017). These results are consistent with our findings, implying that the promotion of bulblet development by ABA is attributed to the increase in auxin content, including IAA and MeIAA contents.

In addition, endogenous GA and ABA may act as inhibitors of bulblet formation, as their contents were significantly decreased during the first three DAT (Figure 3). Except for ABA treatments, the endogenous ABA content did not significantly differ between other treatments (Figure 3), suggesting that endogenous ABA had little effect on regulating bulblet development under different hormone treatments. Furthermore, changes in the contents of four types of endogenous GA showed no significant differences between control, ABA, and ethrel treatments, while endogenous GA_15_ and GA_24_ were strongly inhibited by GA_3_ and PBZ treatments (Figure 3). PBZ is a triazole compound well known for its anti-GA effects due to the inhibition of ent-kaurene biosynthesis, which may decrease GA content in lily bulbs (Zheng et al., 2012). Surprisingly, PBZ treatment increased GA_3_ content from 0 to 3 DAT in the present study (Figure 3), which was consistent with the results obtained in *Amorpha fruticosa* treated with PBZ (Fan et al., 2020). It has been reported that PBZ can increase the expression of GA biosynthesis genes, *GA20ox-3* and *GA3ox2*, through feedback regulation in response to lower GA content caused by PBZ-induced ent-kaurene oxidase inhibition (Hedden and Thomas, 2012). This could partly explain the increase in GA_3_ content after treatment with PBZ.

Carbohydrate metabolism is also important for bulblet development. In our previous study, the starch in the mother scales was served as a carbon source, a source of soluble sugars derived from starch degradation, and a source of sucrose, which were transported to and promoted bulblet formation and development (Xu et al., 2020). When the newly formed bulblets gradually developed, their starch content rose again, which was accompanied by an increase in soluble sugar and sucrose contents. However, in the GA treatment in which bulblets hardly developed, their contents were maintained at a relatively low level compared to those in other treatments (Figures 4A,C,E), implying that carbohydrate accumulation was beneficial for subsequent bulblet development. In addition, we found that starch and soluble sugar contents were the highest in the PBZ treatment after 14 DAT, followed by those in the ethrel and ABA treatments (Figures 4A,C). In lily bulb development, PBZ could improve carbohydrate accumulation, and soluble carbohydrate and starch contents were significantly increased with the increase in PBZ dose (Zheng et al., 2012; Wu et al., 2019). Based on these results, we proposed that GA and PBZ had opposite effects on bulblet development in *L. radiata*, owing to their decreasing and increasing effect on carbohydrate accumulation in bulblets, respectively.

In addition, we found that the GA treatment could significantly inhibit the activities of SUS and AGPase, which belong to sucrose metabolism and starch synthesis enzymes, respectively, but the PBZ treatment had the opposite effect to GA, promoting the activity of these enzymes (Figures 5A,B). Furthermore, GA treatment significantly inhibited the expression levels of *LrSUS1* and *LrSUS2* genes, which encode the SUS enzyme, as well as four genes encoding AGPase large and small subunits (Figures 6, 7). GA had an inhibitory effect on sucrose-induced physiological activities (Loreti et al., 2008), indicating that it inhibited SUS activity and the expression levels of genes encoding SUS. In potato tuberization and tobacco (*Nicotiana tabacum* L.) plant growth process under GA treatments, GA significantly inhibited AGPase activity at both protein and transcriptional levels (Cheng et al., 2018; Manoharlal et al., 2018). The application of the GA biosynthesis inhibitor PBZ could have the opposite effect on GA and promoted the activity and gene expression level of AGPase (Figures 5B, 7). Consistently, uniconazole (another GA biosynthesis inhibitor) could induce starch accumulation in the bioenergy crop duckweed (*Landoltia punctata*), by increasing the activities of key enzymes involved in starch synthesis, as well as the expression levels of these enzymes (Liu et al., 2015). Based on these results, we proposed that GA can inhibit the activities of enzymes involved in sucrose metabolism and starch synthesis through the decrease in the expression levels of genes encoding these enzymes, resulting in slow carbohydrate accumulation and inhibition of bulblet development in *L. radiata*. The application of the GA biosynthesis inhibitor PBZ could decrease the effect of GA on these enzymes, thus promoting bulblet development.

Abscisic acid had a synergic effect with sucrose and enhanced the expression of sucrose-induced genes (Loreti et al., 2008). Similar to our observations in the PBZ treatment, we observed an increase in SUS activity and the expression levels of *LrSUS1* and *LrSUS2* genes in the ABA treatment (Figures 5A, 6). Furthermore, ABA increased the activity of starch synthesis enzymes, SSS and GBSS, by up-regulating the expression of *LrSS1*, *LrSS2*, and *LrGBSS1* genes, which was mainly observed at 7 DAT (Figures 5B,C,D, 8). In rice, ABA was shown to enhance the activities of three key enzymes involved in the sucrose-to-starch pathway in rice grains, SUSase, AGPase, and SSS (Hurkman et al., 2003; Yang et al., 2006). Moreover, in grapevine (*Vitis vinifera* L.) buds, ABA treatment increased the starch content and upregulated the expression of starch synthesis genes *VvSS1* and *VvSS3* (Rubio et al., 2019). These results were all similar to our findings on the effect of ABA on carbohydrate metabolism regulation. In addition, ethylene may be a negative regulator of ABA action (Ghassemian et al., 2000); the application of an inhibitor of ethylene synthesis (cobalt ion) was found to increase the activities of SuSase, AGPase, and SSSase in rice grains, whereas the application of an ethylene-releasing agent (ethephon) decreased their activities (Yang et al., 2006). These results suggested that ethrel application may not directly promote carbohydrate metabolism. We hypothesized that the effect of ethrel on bulblet development may be regulated by wound healing and recovery after cutting, which needs to be verified in future studies.

## Conclusion

In the present study, we tested the effect of different exogenous hormones on bulblet development in *L. radiata*, and found that GA treatment could significantly inhibit bulblet development, whereas PBZ, ABA, and ethrel treatments promoted it. PBZ, a GA biosynthesis inhibitor, particularly promoted bulblet development. GA could inhibit endogenous CK content, which was beneficial for bulblet development. Furthermore, GA could also inhibit the activities of carbohydrate metabolism enzymes, including SUS and AGPase, through the down-regulation of the expression levels of genes encoding these enzymes, including *LrSUS1*, *LrSUS2*, and AGPase large and small subunits, resulting in a decrease in carbohydrate accumulation in the bulblets, thus hindering their subsequent development. PBZ decreased endogenous GA_15_ and GA_24_, and had the opposite effect to GA on carbohydrate metabolism, thus promoting bulblet development. ABA promoted endogenous auxin contents, including IAA and MeIAA, and further promoted the activities of starch synthesis enzymes, especially SSS and GBSS, which may be up-regulated by the expression of *LrSS1*, *LrSS2*, and *LrGBSS1* genes. This could also result in the accumulation of carbohydrates in bulblets and promote their development. In addition, ethrel application could also promote bulblet development in the present study, which could partly be caused by the significant increase in endogenous CK content. Although the accumulation of carbohydrate and starch enzymes was also increased by ethrel, we proposed that the effect was not direct.

## Data Availability Statement

The original contributions presented in the study are included in the article/Supplementary Material, further inquiries can be directed to the corresponding authors.

## Author Contributions

JX, YZ, and YC were responding for designing the work. JX and QL performed the experiments. JX and YL wrote the manuscript. LY contributed to the measurement of physiological indexes and data analysis. All authors contributed to the article and approved the submitted version.

### Conflict of Interest

The authors declare that the research was conducted in the absence of any commercial or financial relationships that could be construed as a potential conflict of interest.

## Footnotes

^1^http://www.metware.cn/

^2^www.shimadzu.com.cn/

^3^www.appliedbiosystems.com.cn/
