# Supplementary material for: Effect of Exogenous Gibberellin, Paclobutrazol, Abscisic Acid, and Ethrel Application on Bulblet Development in Lycoris radiata
Source: Front Plant Sci. 2021 Jan 20;11:615547. doi: 10.3389/fpls.2020.615547 (PMC7855306; doi:10.3389/fpls.2020.615547)
Supplement: Supplementary file 1 [file Table_1.docx]

Supplementary Material

**Supplementary Table 1** | Primers used for qRT-PCR in the present study.

| **Gene ID** | **Gene** | **Forward primer sequence** | **Reverse primer sequence** | **Product length (bp)** |
| --- | --- | --- | --- | --- |
|  |  | **(5'–3')** | **(5'–3')** |  |
| isoform_126867 | *LrSUS1* | TCCTGGGGCTGATATGTCCA | TTTACTCGGTCCAACCTCGC | 180 |
| isoform_27572 | *LrSUS2* | AGCATTTGGGCTCACGGTTA | TGGTAGCCAGAAACGCCATT | 108 |
| isoform_124893 | *LrSUS3* | TCTGGGCTTGGTGATTGTTGA | CTGTGGATGCGAGTCAAAGC | 117 |
| isoform_13 | *LrSUS4* | CACTGGTGGGCAGATTGTCT | CGCTGATTGCATGTAGTGCC | 156 |
| isoform_25827 | *LrAGPL1* | TGGGTGTTCGCTCACGTTTA | TCCCACACCAATTGGAACCT | 131 |
| isoform_128684 | *LrAGPL2* | ATGATGATGGGTGCCGACTC | GAACCCTTCGCTTGGCCTAT | 198 |
| isoform_5076 | *LrAGPS1* | GCTGCAAAAGGTAGCGTTCC | CATCCGTCTCTCTTGCTGCT | 142 |
| isoform_26096 | *LrAGPS2* | TTCGGACTCCAAGAACTCGC | AGCTCCTAAAGGAACCGCTG | 142 |
| isoform_84768 | *LrSS1* | GGCATTCATCGATCCTCCGT | AATCATGGCCACAGAGCCAA | 167 |
| isoform_90712 | *LrSS2* | AACATAGCACACCAGGGTCG | GATGTTTTGAGCTCCCGTGC | 191 |
| isoform_84983 | *LrSS3* | AGCTATTCAAGTCGCGGTCC | AAAGCAGGGCGATTCCATGA | 129 |
| isoform_124644 | *LrGBSS1* | GGCTGATTCAGTTGCAAGGC | TGACAGTTGAGGCGATTGCT | 171 |
|  | *Actin* | CATCCCTCAGCACCTTCCAG | CTGGGATGCAAAAACCGCC | 137 |
